# Microvesicles and inflammatory markers in an animal model of chronic postcapillary pulmonary hypertension

**DOI:** 10.7150/ijms.84924

**Published:** 2023-08-28

**Authors:** Elva Maria Mendoza Zambrano, Belén Gómez Rodríguez, Inés García Lunar, Daniel Pereda Arnau, Verónica Sánchez-López, Ana García Álvarez, Remedios Otero Candelera

**Affiliations:** 1 Instituto de Biomedicina de Sevilla (IBiS). Hospital Universitario Virgen del Ro cio/CSIC/Universidad de Sevilla, Seville, Spain.; 2 Centro de Investigación Biomédica en Red de Enfermedades Respiratorias (CIBERES), Madrid, Spain.; 3 Centro Nacional de Investigaciones Cardiovasculares (CNIC), Madrid, Spain.; 4 CIBER Enfermedades Cardiovasculares (CIBERCV), Madrid, Spain.; 5 Cardiology Department, University Hospital, Madrid, Spain.; 6 Instituto de Investigaciones Biomédicas August Pi i Sunyer (IDIBAPS). Hospital Clinic, Universidad de Barcelona, Barcelona, Spain.

Pulmonary hypertension (PH) is a major global health problem. Postcapillary PH secondary to left heart disease (LHD) is the most prevalent PH subtype. It is characterized by a mean pulmonary artery pressure (mPAP) ≥ 20 mmHg and pulmonary artery wedge pressure (PAWP) heart > 15 mmHg. It is the type of pulmonary hypertension with the worst prognosis, largely due to the lack of an effective treatment 1-4. Vasodilators are the treatment of choice for PH patients. However, they have a limited efficacy in the treatment of endothelial dysfunction of postcapillary PH secondary to LHD 5-8. It is known inflammation is involved in vascular remodeling during PH development 9-12. Specifically, previous reports describe the implication of inteleukin-6 (IL-6) in PH. However, most of them are focused on PH in general terms or only in pulmonary arterial hypertension (PAH) subtype 13, 14. It also well stablished leukocyte-endothelial interaction during the inflammatory process occurs through the interaction of CD44 present on the surface of some leukocytes with glycosaminoglycan hyaluronan, a major component of extracellular matrix 15, 16. Extracellular microvesicles (MVs) are small vesicles derived from different cellular types with a potential role as cellular mediators and effectors. Previous report observed higher levels of different MVs subpopulation like endothelial MVs in PAH 9: Levels of MVs derived from leukocytes expressing CD44+ might be a role in postcapillary PH development although it has not been studied from the moment.

Our aim was to investigate the role of MVs from leukocytes that express CD44+ on their surface and inflammatory mediators like IL-6 in the genesis and development of postcapillary PH in a porcine experimental animal model. The study protocol was approved by our Institutional Animal Research Committee (PA 1411). All animals received humane care in compliance with the Guide for the Care and Use of Laboratory Animals. Briefly, 12 three-month-old Large White pigs were used, 10 of them underwent left lateral thoracotomy to ligate the inferior pulmonary venous drainage (postcapillary PH group) and 2 of them underwent surgery without ligation of pulmonary veins (sham-control group) 17. Hemodynamic parameters were obtained by right heart catheterization through the femoral vein. Blood extraction (10 mL) was carried out at baseline and at 8 and 14 weeks after surgery by central venous cannulation with a 21-gauge needle. Blood was collected with 0.109 M trisodium citrate Vacutainer tubes (BD Diagnostics) (discarding the first 3 mL). Tubes were maintained in a vertical position without agitation, transported to laboratory and processed within 2 h of extraction. MVs rich plasma were obtained by centrifugation as described previously by our group 18. Briefly, blood tubes were centrifuged at 1500 g for 30 min at 4 ºC without brake and MVs rich plasma were collected and stored at -80 ºC for biomarkers quantification. Plasma concentration of IL-6 and CD44+CD45+MVs were determined in 7 and 6 pigs with postcapillary PH secondary to LHD respectively. IL-6 was quantified by ELISA method using porcine IL-6 Quantikine ELISA kit (R&D Systems, Minneapolis, MN, USA). MVs expressing CD45 (leukocyte common antigen) and CD44 (activated leukocyte antigen) were quantified by flow cytometry using Megamix Plus SSC (Biocytex, Marseille, France) as calibrator. Data were expressed as median (25th-75th percentile). Mann Whitney U test and Spearman rho coefficient test were applied as appropriate.

At 8 weeks after surgery, pigs undergoing pulmonary vein ligation presented a significant increase of pulmonary arterial pressure (PAP) compared to control group. This difference was maintained at week 14 after intervention demonstrating the successful induction of postcapillary PH in our animal model (Fig 1A). We assessed the association between MVs population and IL-6 levels with hemodynamics parameters. At week 8 after surgery, we observed a strong and positive correlation between IL-6 and diastolic PAP (Rho: 0.995; p = 0.001, Figure 1B) and mean PAP (Rho: 0.821; p = 0.023, Figure 1C). On the other hand, at 14 weeks a good correlation was found between MVs doubly expressing CD44 and CD45 and diastolic PAP (Rho: 0.943; p = 0.005) (Figure 1D).

Our results showed, in an animal model of postcapillary PH, excellent correlations between the increase in diastolic and mean PAP levels and the increase in IL-6 levels and a specific population of leukocyte MVs. IL-6 is considered an inducer of pulmonary remodeling with an impact in proliferation, angiogenesis, vasoconstriction and inflammation. One of its main targets is the JAK/STAT pathway with a proven role in the development of some types of PH 19. Various studies have related IL-6 and other inflammatory mediators to vascular remodeling in some types of PH, although few have focused specifically on postcapillary PH. Regarding MVs, prior evidence has attributed to specific MVs subpopulation a role in vascular remodeling. It has been observed in experimental models that MVs from leukocyte origin stimulate proinflammatory genes in the endothelial cell and cause endothelial dysfunction 20.

Although postcapillary PH secondary to LHD is the most common type of PH, to date, it does not have an effective treatment. Patients with this type of PH have a poor prognosis and usually are difficult to manage clinically. Studies dedicated on finding new therapeutic targets are essential. In this sense, our results suggest that IL-6 and leukocytes MVs might be of interest and the basis for future studies about inflammatory mechanism in postcapillary. These investigations would help to identify potential new therapeutic targets in postcapillary PH secondary to LHD.

## Figures and Tables

**Figure 1:**
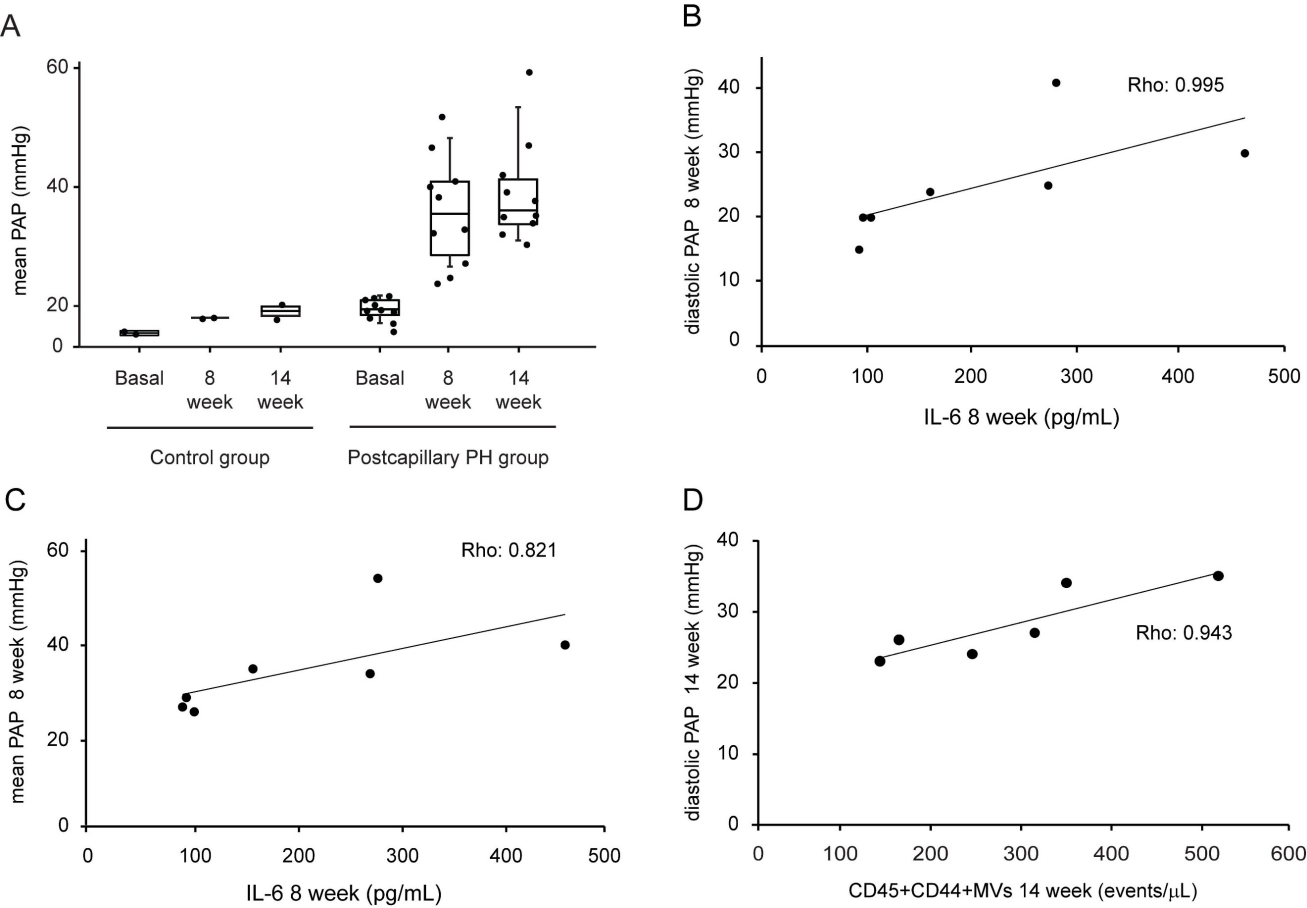
Hemodynamic parameters, IL-6 and MVs levels in postcapillary PH animal model. **A**. Mean PAP levels at basal, 8 week and 14 weeks after intervention in control and postcapillary PH group. **B**. Correlation between diastolic PAP and IL-6 levels at 8 weeks after intervention in postcapillary PH group. **C**. Correlation between mean PAP and IL-6 at 8 weeks after intervention in postcapillary PH group. **D**. Correlation between diastolic PAP and leukocytes MVs at 14 weeks after intervention in postcapillary PH group. PAP: pulmonary arterial pressure; PH: pulmonary hypertension; MVs: microvesicles; IL-6: interleukin 6. Black line: trend line.
